# An effect of the tubular baffles configuration in an agitated vessel with a high-speed impeller on the power consumption

**DOI:** 10.1007/s11696-018-0533-4

**Published:** 2018-06-13

**Authors:** Marta Major-Godlewska, Joanna Karcz

**Affiliations:** Department of Chemical Engineering, West Pomeranian University of Technology, Szczecin, al. Piastów 42, 71-065 Szczecin, Poland

**Keywords:** Vertical tubular baffles, Agitated vessel, High-speed impeller, Non-Newtonian liquid, Power consumption

## Abstract

The results of the power consumption for an agitated vessel equipped with vertical tubular baffles and high-speed impeller are presented in the paper. Aqueous solutions of CMC were agitated within transitional range of the non-Newtonian liquid flow in the agitated vessel of inner diameter equal to 0.6 m. Eight different types of the impellers were tested: Rushton or Smith turbines, turbine with straight blades, pitched blade turbines and propeller. The *J* tubular baffles of outer diameter *B* were located in the position *e* from the vessel wall. Different configurations of baffles, arranged around the vessel circumference singularly or blocked in the modules, were considered in the study. In total, 180 different tubular baffles–impeller systems were tested. The measurements of the torque were conducted by means of the strain gauges method. Based on the power characteristics obtained for each impeller type, the effect of the geometrical parameters of the vertical tubular baffles on the power number was determined and discussed. The results show that geometry of the tubular baffles mostly affects the power number for the system with radial flow Rushton turbine. Moreover, power numbers decrease with the increase of the clearance between baffle and vessel wall for the systems, in which the radially axial circulation of the liquid is promoted. The dependences of the power number on the geometrical parameters of the vertical tubular baffles arranged singularly around the vessel circumference were described by means of the Eqs. (5)–(16). These equations can be useful in the project computations.

## Introduction

Vertical tubular baffles arranged in a vessel (a reactor or bioreactor) can simultaneously fulfil a role of cooling (or heating) the coil in such an apparatus. Heat transfer, especially, in bioreactors with mechanical agitators is a very important problem because of the necessity of accurate control for bioprocess temperature. High requirements are needed for agitating unit (baffles–impeller system) mounted in a bioreactor. There are focused on the effective agitation using possible low specific agitation energy to avoid the microorganisms’ destruction and simultaneously to enable good conditions of the process realization (mass and heat transfer, gas dispersion, etc.).

From the point of view of the bioprocess engineering, it is very important to know that vertical tubular baffles in a bioreactor cause lower shear stresses in a fluid than vertical planar baffles. Large difference in agitation energy requirement is observed for a case of complete hindering of tangential liquid velocity in a baffled vessel (full baffling) compared to an un-baffled vessel. Taking into account these differences, it can be supposed that the best configuration of the geometrical system consisting of tubular baffles–high-speed impeller exists for which the assumed intensity of the transport processes can be obtained. Proper identification of such system should help in energy saving in a bioreactor and simultaneously improve the quality of the agitation. Moreover, the use of such improved agitating system in a bioreactor enables to expect the decrease of the eventual destruction level of the microorganisms in a bioreactor resulting in a gentle agitation.

Power consumption *P* in an agitated vessel depends on different factors such as type of the impeller, geometrical parameters of the baffles–impeller system and range of the fluid flow among others. Power characteristics *Ne* = *f*(*Re*, *Fr*) are presented in the form of the following equation

1 $$ Ne = CRe^{A} Fr^{{B_{0} }} , $$

where *C* denotes coefficient dependent on the geometrical parameters of the agitated vessel, *A* and *B*_o_ are exponents and dimensionless numbers: Newton *Ne*, Reynolds *Re* and Froude *Fr* are defined as follows

2 $$ Ne = \frac{P}{{n^{3} d^{5} \rho }};\quad Re = \frac{{nd^{2} \rho }}{\eta };\quad Fr = \frac{{n^{2} d}}{g} $$

When non-Newtonian pseudoplastic liquid of rheological properties described by means of power law model is agitated then to the definition of Reynolds number *Re*, apparent viscosity *η*_ae_ is introduced as

3 $$ \eta_{\text{ae}} = k_{\text{c}} (\gamma_{\text{e}} )^{{m_{1} - 1}} = k_{\text{c}} (B_{1} n)^{{^{{m_{1} - 1}} }} , $$

where *γ*_e_ denotes mean shear rate, *m*_1_—flow index, *k*_c_—consistency index, *B*_1_—constant dependent on the impeller type (Nagata 1975; Stręk 1981; Harnby et al 1992), *n*—agitator speed. Then, *Re* number has the following form

4 $$ Re = \frac{{nd^{2} \rho }}{{\eta_{\text{ae}} }} = \frac{{n^{{2 - m_{1} }} d^{2} \rho }}{{k_{\text{c}} B_{1}^{{m_{1} - 1}} }} $$

In literature, there is a lot of information on the power consumption of agitated vessels equipped with vertical planar baffles (Nagata 1975; Stręk 1981; Oldshue 1983; Harnby et al 1992; Paul et al 2004). However, the results of the power consumption of the vessel with tubular vertical baffles are not sufficient. The system of the vertical tubular elements was considered by Dunlap and Rushton (1953), Havas et al (1982), as well as Wang and Yu (1989) as heat transfer surface area alternative to the helical coil mounted in the agitated vessel. Man et al (1991) studied an effect of the liquid rheology (Newtonian liquids and non-Newtonian CMC solutions) and geometry of the tubular baffles on the heat transfer process and power consumption to evaluate optimal geometry of the agitated vessel. The measurements were carried out for the standard agitated vessel with Rushton turbine, but the results were not described mathematically. Heat transfer process in the agitated vessel equipped with vertical tubular coil was experimentally studied by Karcz and Major (2001a, b) for non-Newtonian liquid, as well as by Rosa et al (2013, 2017) for Newtonian liquid.

Karcz et al (2001) and Kato et al (2007) experimentally analysed transport phenomena around cylindrical baffles located in the agitated vessel. Gas hold-up and power consumption for the gas–Newtonian liquid system produced in the agitated vessel with tubular baffles were investigated by Major-Godlewska and Karcz (2003, 2011), as well as by Wan et al (2016). Major-Godlewska and Karcz (2012) also experimentally studied process characteristics for the three phase gas–solid–Newtonian liquid system.

Literature survey shows that to date the results of the power consumption for vertical tubular baffles of different geometry and configurations are not completed. The knowledge about power characteristics for such baffles is very important, especially for the non-Newtonian liquid, because energy consumption of the process occurring in the agitated vessel can be estimated on this basis.

The aim of the study presented in this paper was to experimentally determine the effect of the tubular baffles configuration and impeller type on the power consumption required to agitate pseudoplastic liquid within the transitional range of the fluid flow in an agitated vessel.

## Experimental

The experimental investigations were carried out in the agitated vessel of inner diameter *D* = 0.6 m, filled with the non-Newtonian liquid up to the height *H* = *D*. The measurements were performed for the eight types of the high-speed impellers of diameter *d* = 0.33*D*, mounted on the shaft at the distance *h* = 0.33*D* from the flat bottom of the agitated vessel. Aqueous solutions of the carboxymethyl cellulose (CMC) with different concentrations were agitated within the range of the transitional liquid flow.

**Fig. 1:**
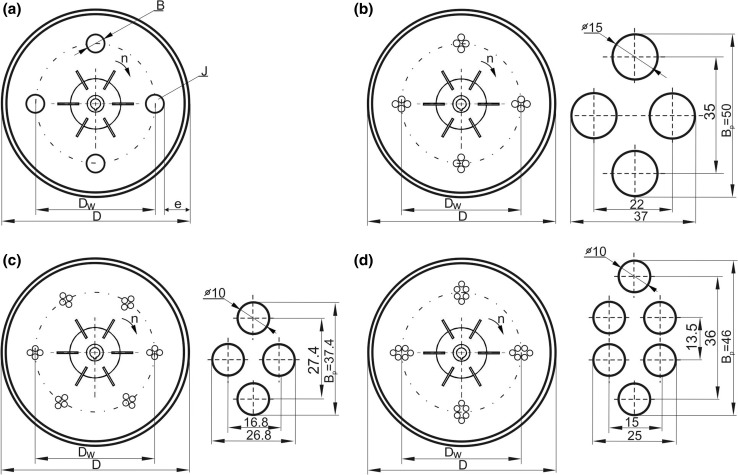
Arrangement of vertical tubular baffles in the agitated vessel; *JB*/*D* = 0.4; **a** *J* = 4, 6, 8, 16 or 24; **b** *J* = 16; *k* = 4; *m* = 4; *B*_p_ = 50 mm; **c** *J* = 24; *k* = 6; *m* = 4; *B*_p_ = 37.4 mm; **d** *J* = 24; *k* = 4; *m* = 6; *B*_p_ = 46 mm

Different systems of the vertical tubular baffles were mounted in the agitated vessel. For each impeller, 23 different configurations of the tubular baffles were tested, which were chosen in this way that the following constraint was fulfilled: product of the baffles number *J* and geometrical parameter *B*/*D* (ratio of the baffle width *B* to the diameter *D*) was equal to 0.4. The value *JB*/*D* = 0.4 corresponds to the state of the *full baffling* in case of the agitated vessel equipped with the standard vertical planar baffles (*J* = 4; *B*/*D* = 0.1). Arrangement of the vertical tubular baffles in the vessel is illustrated in Fig. 1.

Experiments were carried out for the baffles singularly located upon the circumference at different distance *e* from the vessel wall (Fig. 1a). In the case of the *J* = 16 (Fig. 1b) and *J* = 24 baffles (Fig. 1c, d), these baffles were also grouped at the *k* modules, containing *m* = 4 or *m* = 6 baffles in the each module.

In five cases, singular baffles of number *J* = 4, 6, 8, 16 or 24 and baffle width *B* equal to 60, 40, 30, 15 or 10 mm, respectively, were arranged symmetrically around the vessel circumference in the position *e* from the vessel wall (configurations C1–C11 and C15–C17 in Table 1).

**Table 1:** Configuration of the vertical tubular baffles used in the study; fulfilled condition *JB*/*D* = 0.4

No.	*J*	*k*	*m*	*B* (mm)	*B*/*D*	*B*_p_ (mm)	*B*_p_/*D*	*e*/*D*	*D*_w_/*D*
C1	4	4	1	60	0.1			0	0.9
C2	4	4	1	60	0.1			0.1	0.7
C3	6	6	1	40	6.67 × 10^−2^			0	0.93
C4	6	6	1	40	6.67 × 10^−2^			0.042	0.85
C5	6	6	1	40	6.67 × 10^−2^			0.117	0.7
C6	8	8	1	30	0.05			0	0.95
C7	8	8	1	30	0.05			0.05	0.85
C8	8	8	1	30	0.05			0.125	0.7
C9	16	16	1	15	0.025			0	0.975
C10	16	16	1	15	0.025			0.063	0.85
C11	16	16	1	15	0.025			0.138	0.7
C12	16	4	4	15	0.025	50	0.083	0	0.917
C13	16	4	4	15	0.025	50	0.083	0.073	0.792
C14	16	4	4	15	0.025	50	0.083	0.148	0.642
C15	24	24	1	10	1.67 × 10^−2^			0	0.983
C16	24	24	1	10	1.67 × 10^−2^			0.067	0.85
C17	24	24	1	10	1.67 × 10^−2^			0.142	0.7
C18	24	6	4	10	1.67 × 10^−2^	37.4	0.062	0	0.938
C19	24	6	4	10	1.67 × 10^−2^	37.4	0.062	0.076	0.804
C20	24	6	4	10	1.67 × 10^−2^	37.4	0.062	0.151	0.654
C21	24	4	6	10	1.67 × 10^−2^	46	0.077	0	0.923
C22	24	4	6	10	1.67 × 10^−2^	46	0.077	0.084	0.79
C23	24	4	6	10	1.67 × 10^−2^	46	0.077	0.159	0.64
Planar baffles	4			60	0.1			0	

The measurements were performed for three different positions of the tubular baffles inside the vessel, including both limiting positions: directly at the vessel wall (*e* = 0) and the configuration described by the geometrical parameter *D*_w_/*D* ≈ 0.7 (where *D*_w_ is diameter of the tubular baffles system and it corresponds to the helical coil loop diameter). The baffles with numbers *J* = 16 were also grouped at *k* = 4 modules with *m* = 4 baffles in the each module and they were located at three distances from the vessel wall (configurations C12–C14 in Table 1). Moreover, *J* = 24 baffles were grouped at *k* = 6 modules with *m* = 4 baffles or at *k* = 4 modules with *m* = 6 baffles (configurations C18–C23 in Table 1). Therefore, as the data in Table 1 show, the position and configuration of the baffle system are determined by the following geometrical parameters: *D*_w_/*D*, *e*/*D*, *k* (number of modules), *m* (number of baffles in the single module), number of baffles *J* (which is equal to *k*·*m*), as well as *B*/*D* (or *B*_p_/*D*).

**Fig. 2:**
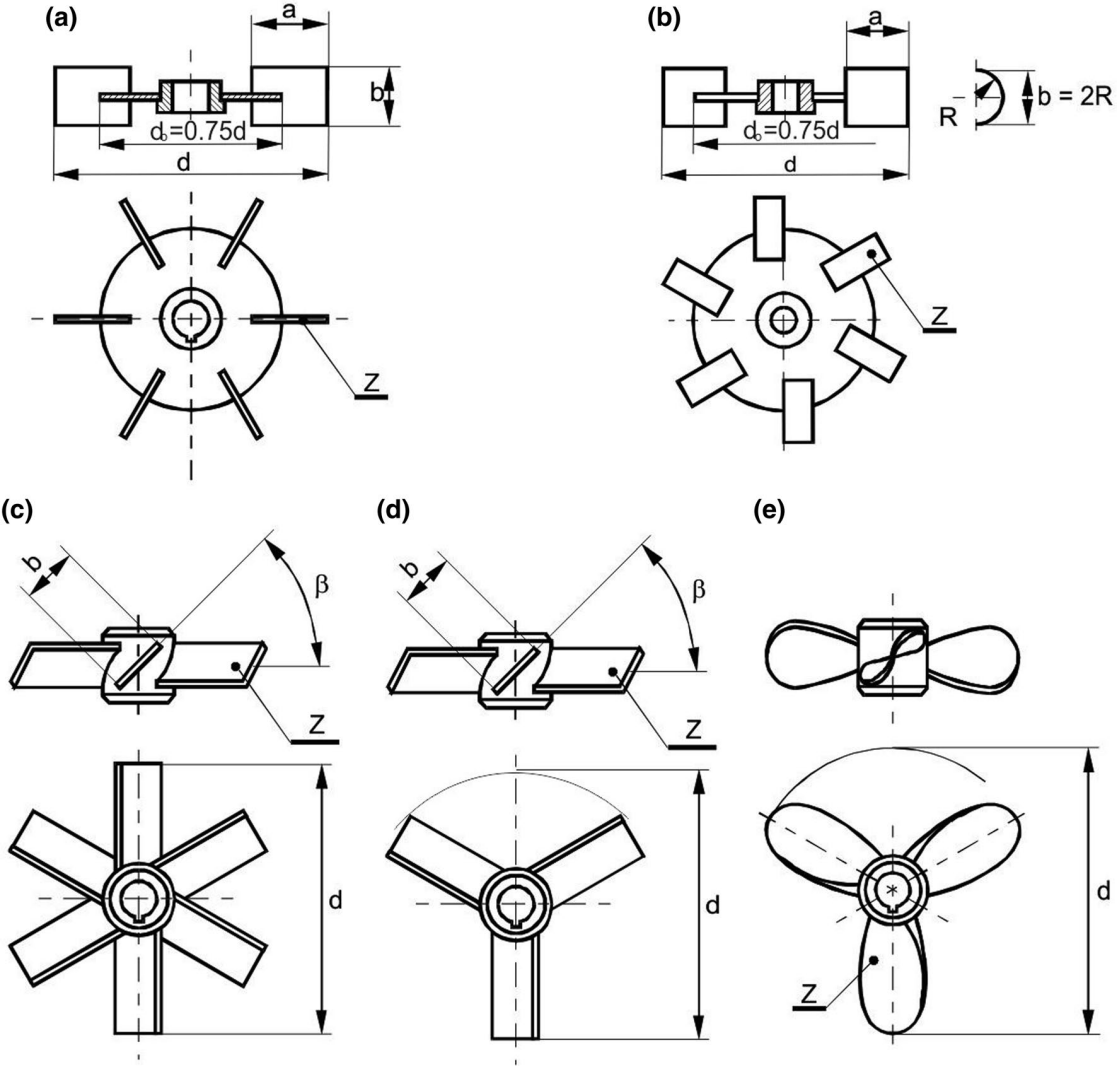
Types of the impellers used; **a** Rushton turbine; **b** Smith turbine (CD 6); **c** pitched blade turbine, *Z* = 6; *β* = 90°; *β* = 60°, 45°; 30°; **d** pitched blade turbine, *Z* = 3; *β* = 45°; **e** propeller

The measurements were carried out for the following eight high-speed impellers of diameter *d* = 0.33*D* shown in Fig. 2: Rushton or Smith turbine, up-pumping pithed blade turbine (PBT) with the *Z* = 6 blades and an angle of the blade inclination *β* equal to 90°, 60°, 45° or 30°, up-pumping PBT with the *Z* = 3 blade and *β* = 45°, as well as up-pumping propeller with *Z* = 3 blades and pitch *S* equal to *d*. Geometrical parameters of the impellers used in the study are collected in Table 2.

**Table 2:** Geometrical parameters of the impellers used in the study

No.	Agitator	*d*/*D*	*Z*	*a*/*d*	*b*/*d*	*β* (deg)	Remarks
1	Rushton turbine	0.33	6	0.25	0.2		
2	Smith turbine	0.33	6	0.25	0.2		*R* = *b*/2
3	Turbine	0.33	6		0.2	90	
4	Pitched blade turbine (PBT)	0.33	6		0.2	60	
5		0.33	6		0.2	45	
6		0.33	6		0.2	30	
7		0.33	3		0.2	45	
8	Propeller, *S*/*d* = 1	0.33	3		0.2^a^		

^a^Max value

Rheological characteristics of two aqueous solutions CMC were determined using rheoviscometer RT 10. The following values of the consistence index *k*_c_ and flow index *m*_1_ for the power law model were found, respectively: *k*_c_ = 0.47 Pa s^m1^, *m*_1_ = 0.35 and *k*_c_ = 0.38 Pa s^m1^, *m*_1_ = 0.51.

**Fig. 3:**
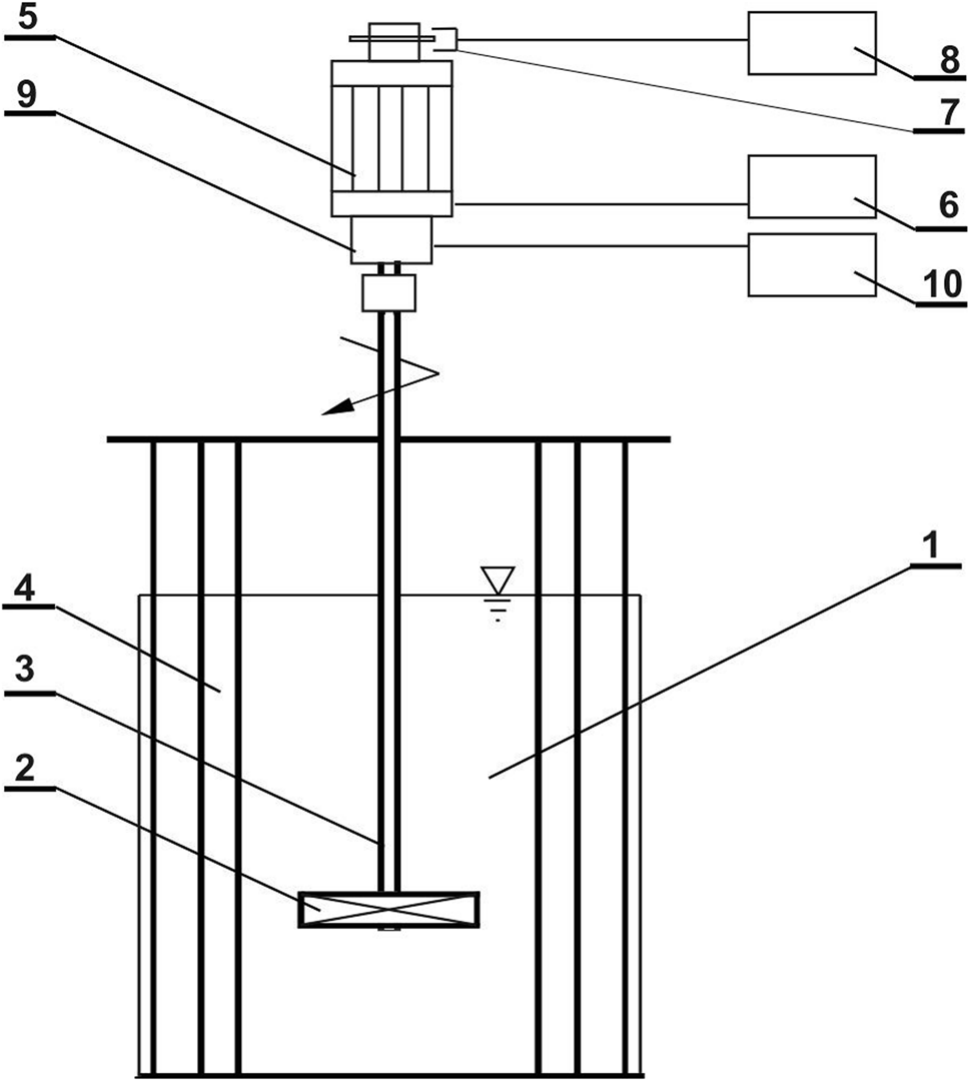
Experimental set-up; 1—agitated vessel; 2—impeller; 3—shaft; 4—vertical tubular baffle; 5—electromotor; 6—steering device; 7—photoelectric sensor; 8—electronic counter; 9—torsional sleeve with strain gauge and slip rings; 10—amplifier MGC

Scheme of the experimental set-up is shown in Fig. 3. The agitated vessel (1) was equipped with the impeller (2) and vertical tubular baffles (4). Torque *M* of the impeller was measured using strain gauge method and speeds of the impeller were determined by means of the photoelectric method. Impeller shaft (3) was driven by an electromotor (5) coupled with the steering device (6). The system of the torsional sleeve with strain gauges and slip rings (9), located between the shaft of the electromotor and the impeller, was connected with the amplifier MGC (10). The measuring system of the impeller speed *n* consisted of the photoelectric sensor (7) and electronic counter (8). Power consumption *P* was determined as follows, *P* = 2π*nM*. For each experimental point (described by a given geometry of the agitated vessel, agitator speed *n* and torque *M*), averaged values of both *n* and *M* values were determined from 6 and 8 readings, respectively, on the electronic counter (8) and amplifier (10).

## Results and discussion

For the geometry of the vessel–impeller–tubular baffle system tested, power characteristics *Ne* = *f*(*Re*) were determined within the transitional (*Re* $\in$ < 7 × 10^2^; 10^4^ >) regime of the non-Newtonian (pseudoplastic) liquid flow in the agitated vessel. An analysis was carried out for the 180 different geometrical configurations of the baffles–impeller, based on the 7200 experimental data.

**Fig. 4:**
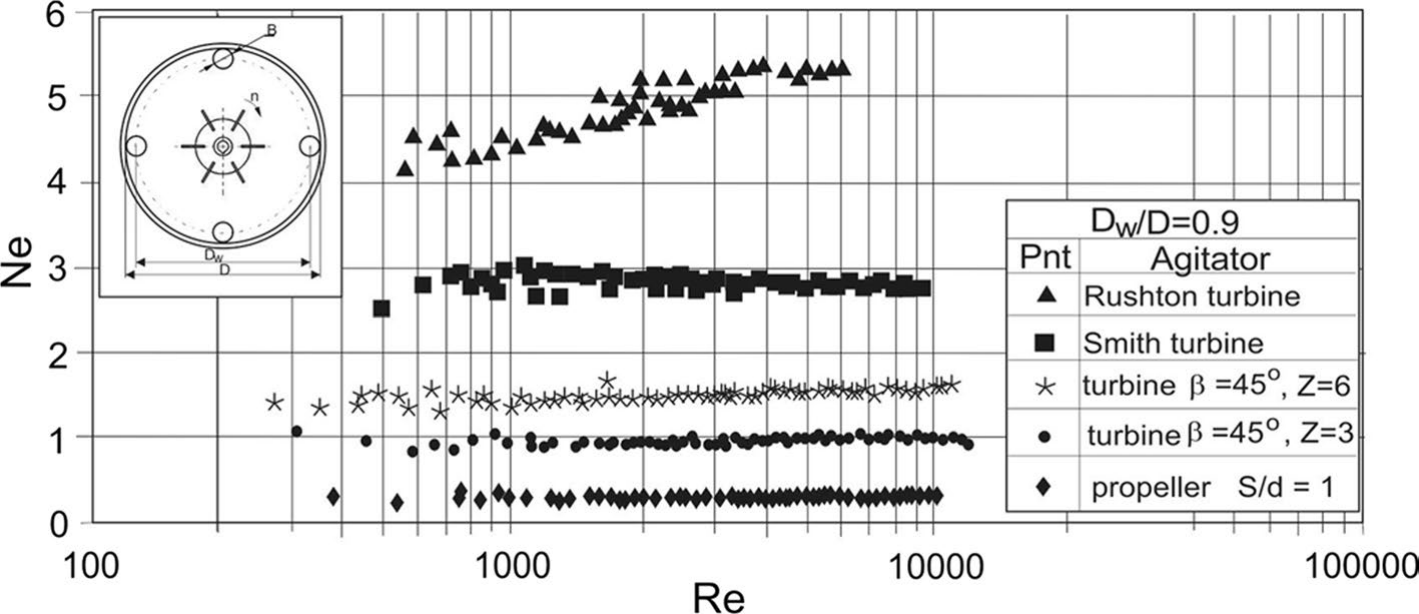
Power characteristics *Ne* = *f*(*Re*) for the agitated vessel with vertical tubular baffles and different impellers; *J* = 4; *B*/*D* = 0.1; *D*_w_/*D* = 0.9; *e*/*D* = 0; liquid: aqueous solution of CMC; baffles configuration C1

**Fig. 5:**
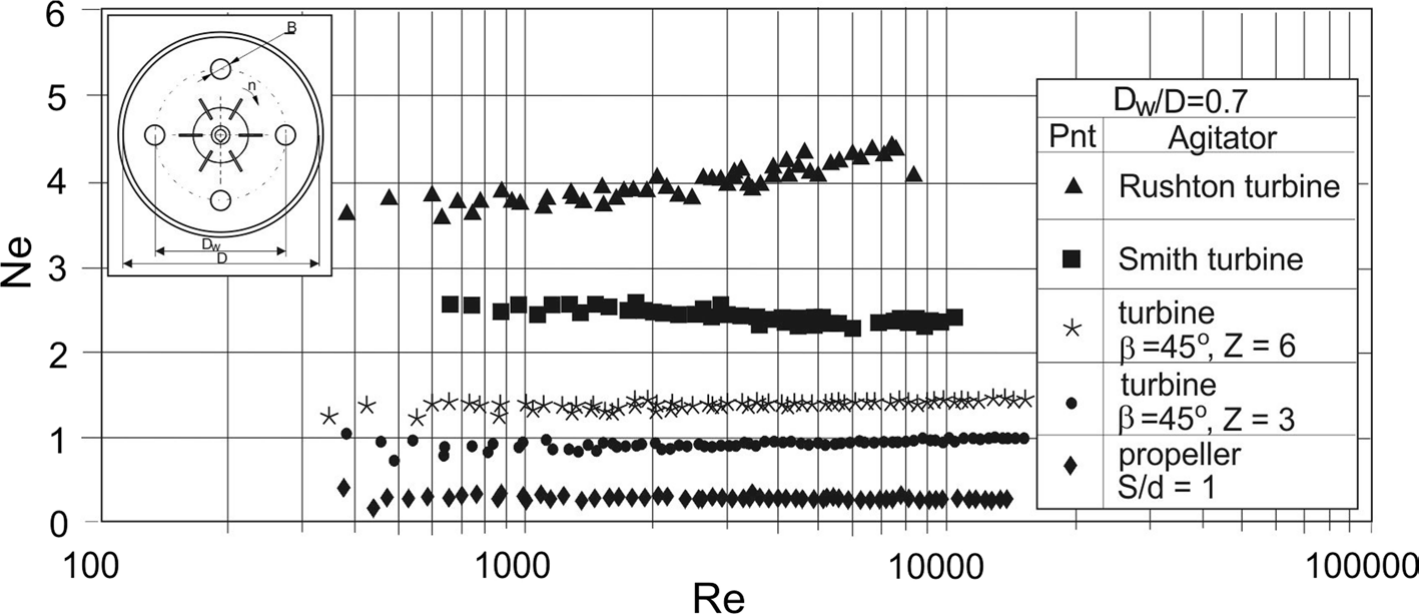
Power characteristics *Ne* = *f*(*Re*) for the agitated vessel with vertical tubular baffles and different impellers; *J* = 4; *B*/*D* = 0.1; *D*_w_/*D* = 0.7; *e*/*D* = 0.1; liquid: aqueous solution of CMC; baffles configuration C2

**Fig. 6:**
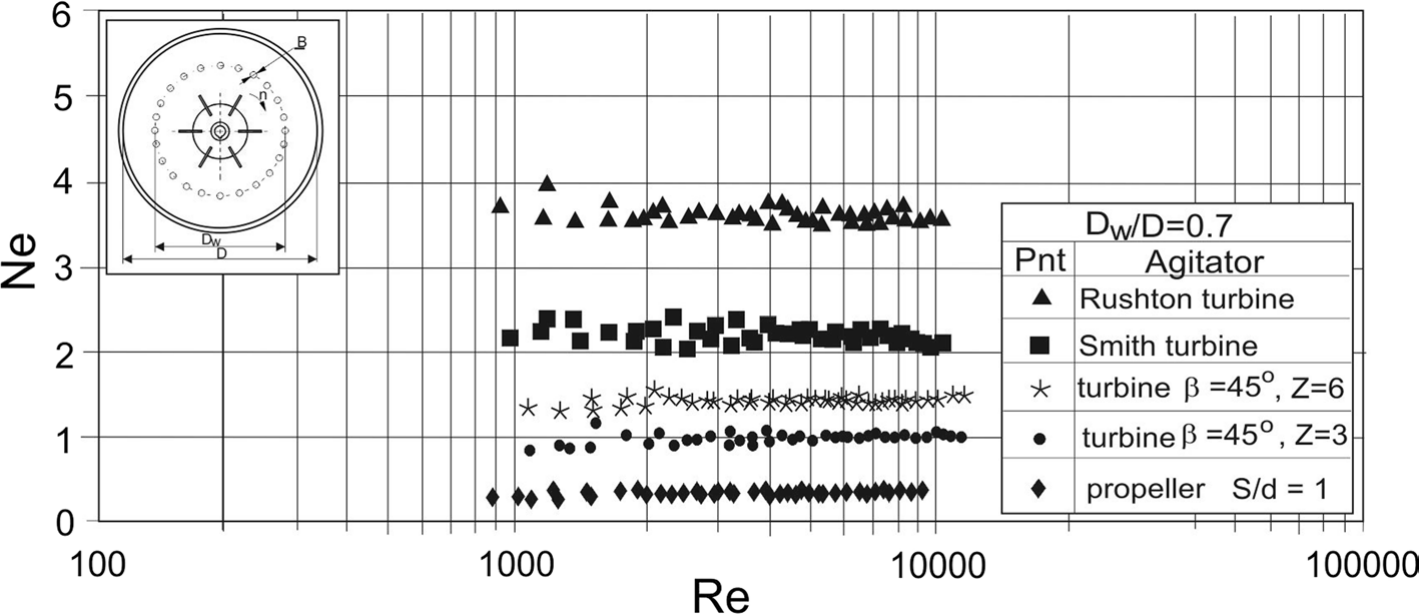
Power characteristics *Ne* = *f*(*Re*) for the agitated vessel with vertical tubular baffles and different impellers; *J* = 24; *B*/*D* = 1.67 × 10^−2^; *D*_w_/*D* = 0.7; *e*/*D* = 0.14; liquid: aqueous solution of CMC; baffles configuration C17

**Fig. 7:**
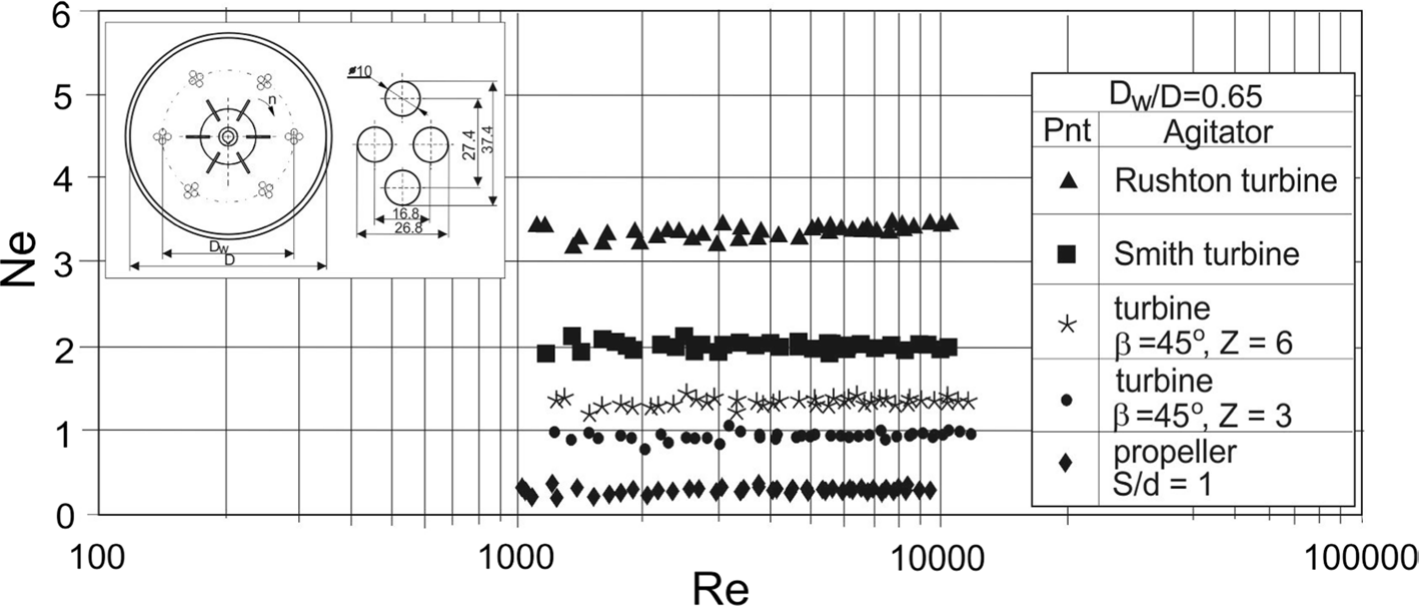
Power characteristics *Ne* = *f*(*Re*) for the agitated vessel with vertical tubular baffles and different impellers; *J* = 24; *k* = 6; *m* = 4; *B*/*D* = 1.67 × 10^−2^; *D*_w_/*D* = 0.65; *e*/*D* = 0.15; liquid: aqueous solution of CMC; baffles configuration C20

Examples of the power characteristics are presented graphically in Figs. 4, 5 and 6, 7 where the results obtained for a given configuration of the vertical tubular baffles cooperating with Rushton or Smith turbines, pitched blade turbines with *Z* = 6 or 3 blades inclined at angle of *β* equal to 45°, or propeller are compared. Figures 4 and 5 illustrate the dependences *Ne* = *f*(*Re*) for the *J* = 4 tubular baffles singularly arranged on the baffle (coil) loop diameter *D*_w_ and high-speed impellers differing in the type of the produced liquid circulation. The highest values of the power number *Ne* were obtained for the tubular baffle systems cooperating with the radial flow Rushton turbine. As Figs. 4 and 5 show power characteristics *Ne* = *f*(*Re*) depend on the Reynolds number *Re* for the system: Rushton turbine—four tubular baffles located immediately of the vessel wall. This effect disappears in the case of the four other impellers (Smith turbine, PBT45 and propeller), especially, when the Reynolds number *Re* is close to value of about 10,000. Comparison of the experimental data *Ne* = *f*(*Re*) in Figs. 4 and 5 shows that the effect of a change of the geometrical parameter *e*/*D* is more visible for the Rushton than for Smith impellers. It is probably caused by modification of the impeller blades shape. Curved blades of Smith turbine affect the mitigation of the radial liquid circulation in the vessel compared to the radial circulation promoted by Rushton turbine and thus requirement of power consumption in an agitated vessel reduces.

Power characteristics *Ne* = *f*(*Re*), representing the configuration of *J* = 24 tubular baffles arranged singularly or blocked in *k* modules with *m* tubes in a module, are shown in Figs. 6 and 7 for different types of the impellers. Comparison of the dependences *Ne* = *f*(*Re*) for those baffles configuration shows that power number noticeable decreases for the agitated vessel with Rushton turbine and tubular baffles combined at the modules compared to *J* singularly arranged tubular baffles.

Figures 6 and 7 show that the effect of the arrangement of the tubular baffles into *k* modules (Fig. 7) instead of singular baffles (Fig. 6) on the power number *Ne* is more visible for the system with Rushton turbine than for the other tested impellers. Probably blocking of the baffles into modules in the system cooperating with the strictly radial flow Rushton turbine mostly decreases the resistance of the liquid flow in the agitated vessel compared to the other impeller–tubular baffles configurations.

Moreover, for the systems with the radial flow impellers, power numbers *Ne* increase with the increase of the baffle (coil) loop diameter *D*_w_.

In the case where power number *Ne*, practically, was not dependent on the Reynolds number *Re*, the values of the *Ne* were averaged for the whole range of the transitional liquid flow in the agitated vessel. Averaged power numbers obtained for both aqueous solutions of CMC and different geometry of the vessel–impeller–tubular baffle system tested are collected in Table 3.

**Table 3:** Power numbers *Ne* for different tested systems of vertical tubular baffles–high-speed impeller

Baffle configuration	Impeller type							
	Rushton turbine *Z* = 6	Smith turbine *Z* = 6	Turbine *β* = 90° *Z* = 6	PBT *β* = 60° *Z* = 6	PBT *β* = 45° *Z* = 6	PBT *β* = 30° *Z* = 6	PBT *β* = 45° *Z* = 3	Propeller *S*/*d* = 1 *Z* = 3
*Ne*								
C1		2.81	4.2		1.50		0.96	0.30
C2	4.01	2.40	3.96		1.41		0.935	0.295
C3	4.92	2.65	4.15	2.43	1.47	0.635	0.955	0.31
C4	3.77	2.31	3.96	2.31	1.42	0.60	0.96	0.325
C5	3.57	2.22	3.65	2.23	1.37	0.625	0.92	0.30
C6	4.60	2.54	4.07	2.38	1.45	0.62	0.94	0.31
C7	3.58	2.20	3.66	2.21	1.40	0.61	0.92	0.305
C8	3.37	2.11	3.72	2.14	1.36	0.59	0.91	0.305
C9	4.62	2.51	4.02	2.33	1.37	0.57	0.915	0.315
C10	3.77	2.26	3.77	2.26	1.43	0.64	0.96	0.325
C11	3.61	2.20	3.69	2.24	1.41	0.64	0.955	0.33
C12	4.87	2.64	4.12	2.43	1.49	0.65	1.02	0.35
C13	3.82	2.31	3.96	2.34	1.46	0.66	1.00	0.335
C14	3.67	2.14	3.79	2.11	1.38	0.57	0.95	0.285
C15	4.02	2.33	3.71	2.30	1.40	0.59	0.96	0.315
C16	3.85	2.32	3.80	2.29	1.45	0.635	1.00	0.345
C17	3.65	2.22	3.69	2.25	1.43	0.625	0.895	0.335
C18	4.46	2.37	4.00	2.19	1.37	0.55	0.94	0.29
C19	3.35	2.04	3.47	2.03	1.33	0.56	0.95	0.26
C20	3.42	2.00	3.47	2.10	1.34	0.56	0.93	0.28
C21	4.71	2.55	4.24	2.43	1.49	0.62	1.00	0.35
C22	3.52	2.13	3.69	2.24	1.44	0.61	0.985	0.32
C23	3.60	2.13	3.71	2.24	1.42	0.60	0.98	0.32
Planar baffles	5.75	2.8	4.35		1.46			0.33

The dependences *Ne* = *f*(*Re*, geometrical parameters) for different high-speed impellers tested and tubular baffles singularly arranged around the vessel circumference (assuming constraint *JB*/*D* = 4) were described mathematically. The Eqs. (5)–(16), approximated with the relative error ± Δ and collected in Table 4, are proposed to evaluate the effect of the impeller type and baffles configuration on the power number *Ne*.

**Table 4:** Equations *Ne* = *f*(geometrical parameters) for different systems of impeller–vertical tubular baffles configuration and transitional flow of the pseudoplastic liquid

No.	Impeller	Equation	No of Eqn.	Range	± Δ, %	Baffle configuration
1	Rushton turbine	*Ne* = 2.516*Re*^0.088^	(5)	*e*/*D* = 0; *JB*/*D* = 0.4; *J* = 4; *Re* $\in$ < 330; 4500)	± 8	C1
2	Rushton turbine	*Ne* = 5.34	(6)	*e*/*D* = 0; *JB*/*D* = 0.4; *J* = 4; *Re* $\in$ < 4500; 6000)	± 3	C1
3	Rushton turbine	*Ne* = 4.01	(7)	*D*_w_/*D* = 0.7; *J* = 4; *JB*/*D* = 0.4	± 4	C2
4	Rushton turbine	*Ne* = 5.13(*J*/4)^−0.12^	(8)	*e*/*D* = 0; *JB*/*D* = 0.4; 6 < *J* < 24	± 4	C3, C6, C9, C15
5	Rushton turbine	*Ne* = 3.65	(9)	*JB*/*D* = 0.4; 6 < *J* < 24; 0.7 < *D*_w_/*D* < 0.85	± 8	C4, C5, C7, C8, C10, C11, C16, C17
6	Smith turbine (CD 6)	*Ne* = 2.768(*J*/4)^−0.09^	(10)	*e*/*D* = 0; *JB*/*D* = 0.4; 4 < *J* < 24	± 4	C1, C3, C6, C9, C15
7	Smith turbine (CD 6)	*Ne* = 2.247	(11)	*JB*/*D* = 0.4; 4 < *J* < 24; 0.7 < *D*_w_/*D* < 0.85	± 6.5	C2, C4, C5, C7, C8, C10, C11, C16, C17
8	Turbine Z = 6 *β* = 90°	*Ne* = 4.24(*J*/4)^−0.06^	(12)	*e*/*D* = 0; *JB*/*D* = 0.4; 4 < *J* < 24	± 4	C1, C3, C6, C9, C15
9	Pitched blade turbine (PBT) *Z* = 6 30° < *β* < 60°	*Ne* = 3.346(sin*β*)^2.47^	(13)	*e*/*D* = 0; *JB*/*D* = 0.4; 4 < *J* < 24	± 4	C1, C3, C6, C9, C15
10	Pitched blade turbine (PBT) *Z* = 6 30° < *β* < 60°	*Ne* = 3.50(sin*β*)^2.54^	(14)	*JB*/*D* = 0.4; 4 < *J* < 24; 0.7 < *D*_w_/*D* < 0.85	± 8	C2, C4, C5, C7, C8, C10, C11, C16, C17
11	Pitched blade turbine (PBT) *Z* = 3 *β* = 45°	*Ne* = 0.949	(15)	*JB*/*D* = 0.4; 4 < *J* < 24; 0.7 < *D*_w_/*D* < 0.983	± 4	C2, C4, C5, C7, C8, C10, C11, C16, C17
12	Propeller	*Ne* = 0.298(*J*/4)^0.061^	(16)	*JB*/*D* = 0.4; 4 < *J* < 24; 0.7 < *D*_w_/*D* < 0.983	± 4	C2, C4, C5, C7, C8, C10, C11, C16, C17

Equation (5) describes power characteristics for the agitated vessel with Rushton turbine and *J* = 4 baffles of the width *B* = 0.1*D* arranged immediately at the vessel wall (*e*/*D* = 0). In this case, power characteristics depends on the *Re* number within the range of the *Re* number from 330 to 4500. Above *Re* > 4500 power number *Ne* reaches constant value equal to 5.34 (Eq. (6), *Re* $\in$ < 4500; 6000 >). Lower value of the *Ne* than that, but also constant (*Ne* = 4.01) is characteristic for the baffles configuration (*J* = 4; *B*/*D* = 0.1) arranged more closely of the impeller (Eq. (7); *D*_w_/*D* = 0.7). The effect of the baffles number *J* located immediately at vessel wall (*e*/*D* = 0) on the power number *Ne* was revealed only in such a case when baffles number is higher than *J* = 4 and it is contained within the range 6 < *J* < 24 (Eq. (8)). Equation (9) shows that this effect can be neglected when the tubular baffles will be replaced in the direction of the impeller (*D*_w_/*D* $\in$ < 0.7; 0.85 >).

Power number *Ne* for the Smith turbine (CD 6)–tubular baffles system is described using Eqs. (10) (*e*/*D* = 0) and (11) (0.7 < *D*_w_/*D* < 0.85). Similar to the baffles configuration with Rushton turbine, in this case, power number *Ne* depends on the baffles number *J* only for the baffles located immediately at vessel wall (Eq. (10)) and it does not depend for the baffles system replaced towards rotating impeller (Eq. (11)).

Power number *Ne* depends on the baffles number *J* also for the six bladed turbine with straight blades (*β* = 90°)–tubular baffles system [Eq. (12)] when the baffles are arranged immediately at vessel wall (*e*/*D* = 0). In this case, the values of the power number are lower than those for the Rushton turbine (Eq. (8)) but higher than those for the Smith turbine [Eq. (10)]. Absolute value of the exponent at the geometrical parameter (*J*/4) obtained in Eq. (12) for the system with turbine with straight blades is 2 and 1.5 times lower than those ascribed to the system with Rushton [Eq. (8)] and Smith [Eq. (10)] turbines, respectively.

As the Eqs. (13) and (14) show, power numbers *Ne* for the agitated vessel equipped with tubular baffles and up-pumping pitched blade turbine with six blades very strongly depend on the angle inclination *β*, whereas the effect of baffles geometry (number of baffles *J* and baffle loop diameter *D*_w_) can be neglected.

Within the range of the performed experiments (4 < *J* < 24; 0.7 < *D*_w_/*D* < 0.983), geometrical parameters of the tubular baffles do not affect the power number *Ne* in the vessel with the up-pumping pitched blade turbine with *Z* = 3 blades of the angle inclination *β* = 45° [Eq. (15)], but small effect of the baffles number *J* reveals in the case of the system with the up-pumping three bladed propeller (Eq. (16)).

Power numbers *Ne* obtained for the vertical tubular baffles and transitional range of the non-Newtonian liquid (CMC solutions) can be compared with the *Ne* numbers given for the four standard planar baffles (*J* = 4; *B*/*D* = 0.1; *e*/*D* = 0; last line of the Table 3) and turbulent range of the Newtonian liquid flow. Considering baffles configuration C1 with *e*/*D* = 0 and Eq. (6) for the Rushton turbine, as well as the data from Table 3 for the Smith turbine, turbine with straight blades, PBT45° and propeller, small differences are observed only between the *Ne* values for both geometrical systems with tubular and planar baffles within both transitional and turbulent regimes.

**Fig. 8:**
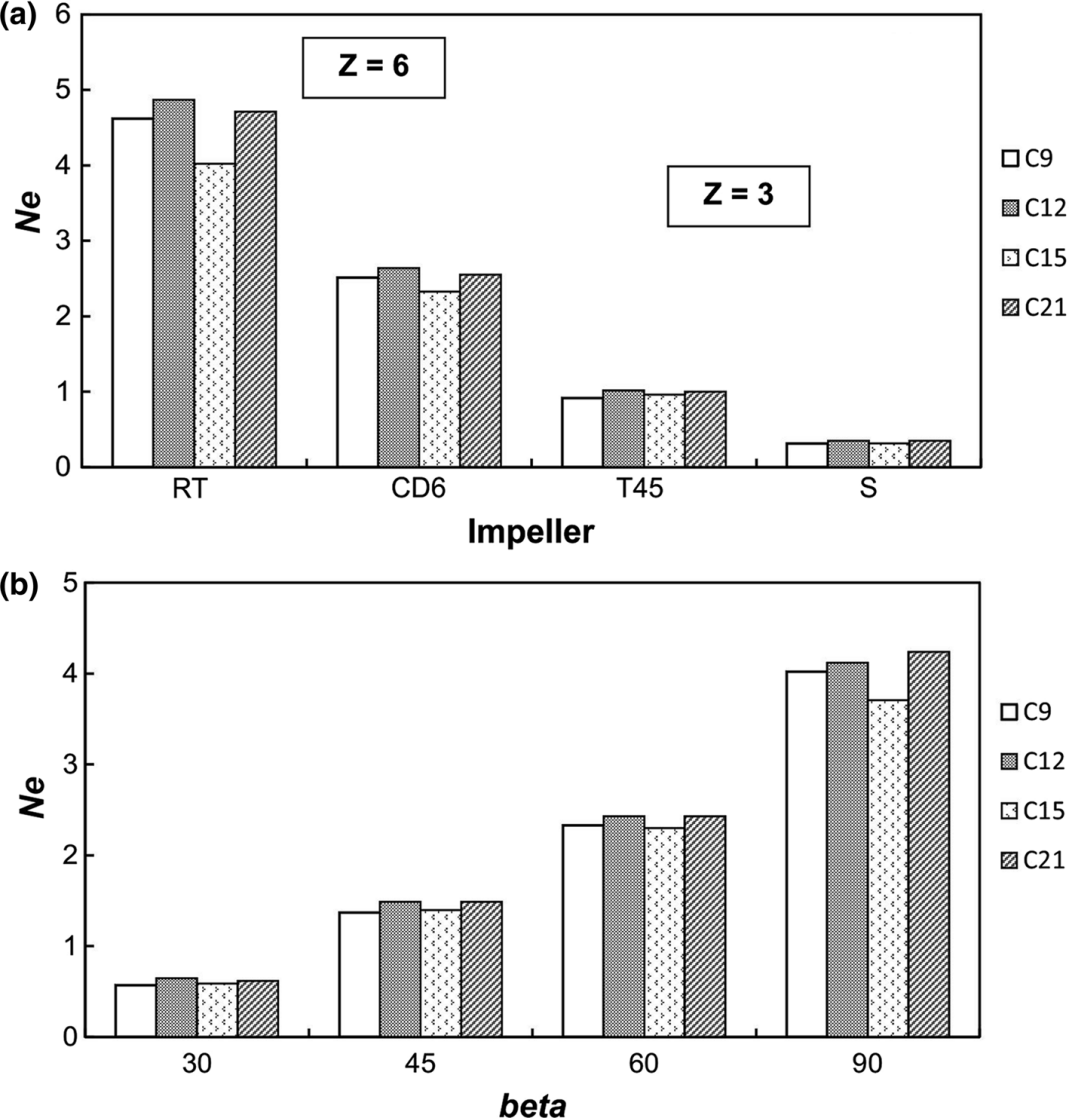
Comparison of the power number *Ne* for different configurations tubular baffles–impeller; *e*/*D* = 0; **a** different impeller types; **b** different pitch *β* of the PBT impeller

Power numbers *Ne* for different tested configurations of tubular baffles–impeller are compared in Fig. 8. These results describe the data for constant value *e*/*D* = 0 and two modes of arrangement of baffles: singularly located (C9 and C15) or blocked (C12 and C21). The lower values of the parameter *D*_w_/*D* correspond to mode of the blocked tubular baffles. The data in Fig. 8a show that, for each tested impeller, power number *Ne* is higher for the system with blocked baffles compared to singularly arranged baffles. Analogous relation is observed in Fig. 8b, where power numbers are compared for baffles configurations C9, C12, C15 and C21 and different pitch *β* of pitched blade turbine.

## Conclusions

The systematic, extensive experimental study was carried out for non-Newtonian liquid (CMC solutions) to evaluate the effect of geometrical parameters of the tubular vertical baffles on the power number in the agitated vessel of inner diameter *D* = 0.6 m, equipped with one of the eight high-speed impellers. An analysis of the power characteristics *Ne* = *f*(*Re*) performed, in total, for 180 different tubular baffles–impeller systems shows that within the transitional liquid flow in the agitated vessel:

1. In the tubular baffles–impeller system, type of the high-speed impeller significantly affects the value of the power number *Ne*. Taking into account energy requirement, the most energy consuming are radial flow impellers (in due order from the highest to lowest value: Rushton or Smith turbine, turbine with straight blades), whereas axial flow propeller is the most energy saving impeller. Mixed flow pitched blade turbines (PBT) are located between both mentioned impeller types and their power requirement decreases with the decrease of the pitch *β* of the impeller blade.
2. In the tubular baffles–impeller system, configuration of the vertical tubular baffles for a given radial flow impeller type is responsible for the diminishing of the baffling effect on the power number with the increase of the clearance between vessel wall and baffles. In case of the axial flow impeller, this effect is not observed.
3. Geometry of the vertical tubular baffles mostly affects the power number *Ne* for the system with Rushton turbine.
4. Power number *Ne* decreases with the decrease of the parameter *D*_w_/*D* for the systems, in which the radially axial circulation of the liquid is promoted.
5. In comparison with the power numbers for the single vertical tubular baffles located symmetrically around the vessel circumference, higher power numbers are obtained for the configuration with the *k* modules of baffles, for assumed number of baffles *J* = const.
6. The results of the power consumption as a function of *Re* number and geometrical parameters of the vertical tubular baffles arranged singularly around the vessel circumference were described by means of the Eqs. (5)–(16), useful in the project computations.

## List of symbols

*A*, *B*_0_: Exponents in Eq. (1)
*a*: Length of the impeller blade (m)
*B*: Width of the baffle (outer diameter of the tubular baffle) m
*B*_p_: Dimension of the baffle module (according to Fig. 1) (m)
*B*_1_: Coefficient in Eq. (3), depending on the impeller type
*b*: Width of the impeller blade (m)
*C*: Constant in Eq. (1)
*D*: Inner diameter of the agitated vessel (m)
*D*_w_: Diameter of the tubular baffles system (m)
*d*: Diameter of the impeller (m)
*e*: Clearance between tubular baffle and wall of the agitated vessel (m)
*Fr*: Froude number (= *n*^2^*d*/*g*)
*g*: Gravitational acceleration (constant) (m s^−2^)
*H*: Liquid height in the vessel (m)
*h*: Distance between impeller and bottom of the vessel (m)
*J*: Number of baffles
*k*: Number of baffle modules
*k*_c_: Consistency index (Pa s^m1^)
*M*: Torque (Nm)
*m*: Number of baffles in the single module
*m*_1_: Flow index
*n*: Impeller speed (s^−1^)
*Ne*: Power (Newton) number (= *P*/(*n*^3^*d*^5^*ρ*))
*P*: Power consumption (W)
*R*: Radius of the impeller blade (m)
*Re*: Reynolds number defined by Eq. (2) or (4)
*S*: Pitch of the propeller blade (m)
*Z*: Number of the impeller blades

## Greek letters

*β*: Angle of the impeller blade inclination to the horizontal deg
*γ*_e_: Effective shear rate (s^−1^)
*η*: Dynamic viscosity of the liquid (Pa s)
*η*_ae_: Effective apparent viscosity (Pa s)
*ρ*: Density of the liquid (kg m^−3^)
